# Seasonal Influenza Vaccine: Uptake, Attitude, and Knowledge Among Patients Receiving Immunoglobulin Replacement Therapy

**DOI:** 10.1007/s10875-020-00922-3

**Published:** 2021-01-05

**Authors:** Fionnuala Cox, Catherine King, Anne Sloan, David J. Edgar, Niall Conlon

**Affiliations:** 1 Department of Immunology, St. James’s Hospital, Dublin 8, Ireland; 2 Department of Immunology, School of Medicine, Trinity College Dublin, Dublin, Ireland

**Keywords:** Influenza vaccine, Immunoglobulin replacement therapy, IVIg, SCIg, Hypogammaglobulinemia

## Abstract

Influenza is a potential cause of severe disease in the immunocompromised. Patients with hypogammaglobulinemia, in spite of adequate replacement therapy, are at risk of significant morbidity and adverse outcomes. A seasonal vaccine is the primary prophylactic countermeasure to limit disease. The aim of this study was to evaluate the attitude, knowledge, and influenza vaccine uptake among Irish patients receiving immunoglobulin replacement therapy (IgRT), as well as uptake in co-habitants. Fifty-seven percent of patients receiving IgRT at a regional immunology referral center completed a questionnaire evaluation. Seventy-six percent of IgRT patients received the influenza vaccine for the 2019 season. Ninety-eight percent recognized that influenza could be prevented with vaccination, and 81% deemed it a safe treatment. Ninety-three percent correctly identified that having a chronic medical condition, independent of age, was an indication for vaccination. Despite excellent compliance and knowledge, many were not aware that vaccination was recommended for co-habitants, and only 24% had full vaccine coverage at home. Those who received advice regarding vaccination of household members had higher rates of uptake at home. This study demonstrates awareness and adherence to seasonal influenza vaccine recommendations among patients receiving IgRT. Over three quarters felt adequately informed, the majority stating physicians as their information source. We identified an easily modifiable knowledge gap regarding vaccination of household members. This data reveals a need to emphasize the importance of vaccination for close contacts of at-risk patients, to maintain optimal immunity and health outcome.

## Introduction

Seasonal influenza is a transmissible acute respiratory illness that causes a mild infection in the majority of people. It is a public health concern as high-risk populations have an increased likelihood of severe disease [1, 2]. There are year-to-year minor antigenic drift and periodic major antigenic shift; thus, annual vaccination is required and has been shown to effectively reduce morbidity and mortality [3, 4].

Infection-related complications are more prevalent in immunocompromised individuals, those over 65 years of age, people with chronic health conditions, and pregnant women [5–8]. The World Health Organization (WHO) estimates over 40,000 people in the European Union die prematurely each year from influenza-related complications, and this figure can be reduced if at least 75% vaccine coverage is attained in risk groups [9, 10]. Disease surveillance is undertaken in most developed countries; however, many nations, including Ireland, have no central registry for vaccine monitoring [11, 12].

Patients with hypogammaglobulinemia are particularly susceptible to disease affecting the respiratory tract [13]. Regular immunoglobulin replacement therapy (IgRT), intravenous or subcutaneous, is the mainstay of management for these patients. Pooled immunoglobulin provides protective titers to most common vaccine agents (mumps, rubella, varicella); however, protection against influenza is ineffective due to antigenic drift [14, 15]. Patients can glean some protective benefit from vaccination, despite impaired ability to develop antibodies; thus, annual vaccination is recommended [16, 17]. In this setting, vaccination of close contacts is vital to limit transmission. Irish and most international guidelines specifically advise vaccination for healthcare workers, carers, and co-habitants of at-risk populations [18–20].

International data demonstrated that failure of patients to identify as “at-risk” as well as perceived cost of vaccination are negative determinants for compliance [21, 22]. Of concern, Irish population-based figures demonstrated a disappointing 28% uptake in those < 65 years with a chronic medical condition, while a 60% uptake in individuals ≥ 65 years still falls short of WHO target coverage [21]. Similar low rates of vaccine uptake are reflected in population-based studies of many developed nations [23, 24].

Global data consistently report suboptimal vaccine uptake in adults with chronic medical conditions; however, there is scant information available on vaccine compliance in those with immunodeficiency disorders [25]. Those with hypogammaglobulinemia are specifically vulnerable to infectious complications leading to hospital admission and adverse health outcomes [17]. Understanding influenza vaccine coverage and attitudes toward vaccination in this group is of particular importance to tailor education and identify specific barriers.

The objectives of this study were to measure seasonal influenza vaccine uptake among patients receiving regular IgRT in our center and to explore knowledge and attitudes toward the influenza vaccine in this population.

## Methods

Information was gathered from November 2019 to March 2020, among adults receiving regular IgRT under the care of the Immunology department in a large Irish tertiary referral hospital. A previously validated questionnaire was used with minor modifications ([22] Appendix). The questionnaire covered four domains: demographics, vaccination and health status, knowledge and education, and co-habitant vaccination. All home-based and hospital day ward–based patients receiving IgRT were invited to take part. Vaccination was not provided by the Immunology department; thus, the data reflects patients receiving vaccination in the community.

Responses were anonymized. The study design and questionnaire were approved by the local Research Ethics Committee (Appendix). Descriptive statistics and chi-squared analysis were applied where relevant and statistical significance set at *p* < 0.05.

## Results

The response rate to the questionnaire was 57% (55/97). Socio-demographic characteristics are detailed in Table 1. The mean age of participants was 51.5 years (22–80 years), and there was a slight male predominance (56%; Table 1). Education status was high among participants; 62% had a tertiary level qualification, and 27% had completed secondary level studies. A sizable portion, 67%, had access to a medical or general practitioner (GP) visit card, thus are eligible for free public health services in Ireland, compared with approximately 30% of the general population ([26]; Table 1). Granting of a medical/GP card is primarily based on income grounds, or if the burden of healthcare costs would result in hardship.

**Table 1:** Demographics of study participants. Mean age was 51.5 years, and 56% were male. Educational attainment was high, but this did not significantly impact on vaccine status. Access to a medical/GP card was associated with increased vaccine uptake, which was independent of solely having access to free vaccination. Chi-squared results are represented by *p* values, *p < 0.05

	Number (%)	Vaccination		Chi-squared
		Yes (%)	No (%)	
Age				
18–49	28 (51%)	21 (75%)	7 (25%)	0.58
50–64	13 (24%)	9 (69%)	4 (31%)	
> 65	14 (25%)	12 (86%)	2 (14%)	
Gender				
Male	31 (56%)	20 (65%)	11 (35%)	0.015*
Female	24 (44%)	22 (92%)	2 (8%)	
Education level				
Primary	4 (7%)	4 (100%)	0	0.4688
Secondary	15 (27%)	12 (80%)	3 (20%)	
Tertiary	34 (62%)	25 (73%)	9 (27%)	
Unanswered	2 (4%)			
Healthcare				
Medical/GP card	37 (67%)	32 (86%)	5 (14%)	0.0074*
Neither	17 (31%)	9 (53%)	8 (47%)	
Unanswered	1 (2%)			
Cost of vaccine				
Access to free Vaccine.	41 (75%)	33 (80%)	8 (20%)	0.22
Pay for vaccine	14 (25%)	9 (64%)	5 (36%)	

In total, 76% participants received the influenza vaccine for the 2019 season, meeting WHO target coverage rate ([25]; Table 2). Levels of vaccination were similar throughout age distributions. There was a notable sex difference, 22 of 24 female patients (92%) being vaccinated compared with 20 of 31 males (65%) (chi-squared < 0.05; Table 1). Vaccine uptake was marginally lower in the preceding years (Table 2). There was consistency with the same patients not engaging in vaccination; however, true rates could be clouded by recall bias.

**Table 2:** Vaccination and health status. In the 2019/20 season, 76% of participants received the influenza vaccine. Numbers were lower in preceding years, as was recall. The majority of participants were not concerned about contracting influenza and reported positively on health status

**Influenza vaccination**			
**Year**	**Yes (%)**	**No (%)**	**Unsure (%)**
2019	42 (76%)	13 (24%)	0
2018	39 (71%)	15 (27%)	1 (2%)
2017	37 (67%)	13 (24%)	5 (9%)
2016	38 (69%)	11 (20%)	6 (11%)
2015	31 (56%)	12 (22%)	12 (22%)
**Perception of Health: 1 bad, 10 very good**			
**Self-reported score**	***N*** **(%)**		
10–7	38 (69%)		
6–4	12 (22%)		
3–1	5 (9%)		
**Hospital admission in the last 12 months**			
**Amount**	**Total**	**Infective cause**	**Non-infective cause**
0	37 (67%)	n/a	n/a
1	10 (18%)	6 (60%)	4 (40%)
> 1	8 (15%)	6 (75%)	2 (25%)
**Concern about developing influenza: 1 not worried, 10 very worried**			
**Self-reported score**	***N*** **(%)**		
10–7	15 (69%)		
6–4	19 (9%)		
3–1	20 (22%)		
Unanswered	1 (2%)		
**Usefulness of influenza vaccine: 1 not useful; 10 very useful**			
**Self-reported score**	***N*** **(%)**		
10–7	43 (78%)		
6–4	11 (20%)		
3–1	0		
Unanswered	1 (2%)		
**Danger of influenza vaccine: 1 not dangerous, 10 very dangerous**			
**Self-reported score**	***N*** **(%)**		
10–7	6 (11%)		
6–4	4 (7%)		
3–1	45 (82%)		
Unanswered	1 (2%)		

Most patients (67%; Table 3) had previously received information regarding the seasonal influenza vaccine. The majority (78%) specified that information was given by healthcare staff. Within this group, various media channels were stated as a secondary information source. For the most part, patients found previous information helpful; 62% reported it as good or very good, while the remaining 30% reported it as moderately useful (Table 3). Ten of 13 unvaccinated patients elaborated on reasons for non-vaccination. The stated reasons included the incorrect belief that vaccination was redundant in those with hypogammaglobulinemia, that vitamin supplements gave sufficient protection, a lack of concern about contracting influenza, or being too unwell to receive it. Association between receiving information and vaccine uptake fell short of statistical significance (chi-squared *p* = 0.09; Table 3). However, interpretation and integration of healthcare advice  is challenging to accurately quantify.

**Table 3:** Vaccine-associated knowledge and education. The majority of participants correctly identified vaccine-preventable disease and populations at increased risk of severe disease from influenza. Most had received information about the influenza vaccine before, which they found useful, and healthcare professionals were stated as the primary source. Chi-squared results are represented by *p* values

Can vaccination protect against infection?	**Yes (%)**	**No (%)**	**Uncertain**	
	47 (85%)	3 (5%)	5 (10%)	
**What diseases are vaccine-preventable?**				
**Disease**	***N*** **(%)**			
Influenza	54 (98%)			
Pneumonia	34 (62%)			
Meningitis	27 (49%)			
AIDS/HIV	2 (4%)			
Heart attack	0			
Common cold	5 (9%)			
**Who is at-risk of severe influenza?**				
**Population**	**Yes (%)**	**No (%)**	**Uncertain (%)**	
Healthy 5–18 year olds	10 (18%)	45 (82%)	5 (9%)	
< 65 years with chronic disease	51 (93%)	4 (7%)	0	
Healthy young adults	5 (9%)	50 (91%)	0	
> 65 years old	51 (93%)	4 (7%)	0	
Pregnant women	18 (33%)	35 (63%)	2 (4%)	
**Influenza awareness**				
**Statement**	**Agree (%)**	**Disagree**	**Uncertain**	
Influenza is rare.	3 (5%)	46 (84%)	6 (11%)	
Influenza is serious.	44 (80%)	2 (4%)	9 (16%)	
Influenza may be prevented.	38 (69%)	5 (9%)	12 (22%)	
**Education regarding the influenza vaccine**				
	** Received vaccination, 2019**			
**Previously received information**	***N*** **(%)**	**Yes (%)**	**No (%)**	**Chi-squared**
Yes	37 (27%)	30 (81%)	7 (19%)	0.093
No	18 (33%)	12 (67%)	6 (33%)	
From a healthcare worker	29 (78%)	24 (88%)	5 (17%)	
From a non-healthcare source	5 (14%)	4 (80%)	1 (20%)	
Not stated	3 (8%)			
**Usefulness of information provided**		**Yes**	**No**	**Chi-squared**
Very good–good	34 (62%)	27 (79%)	7 (21%)	0.33
Moderate	15 (27%)	10 (67%)	5 (33%)	
Bad–very bad	0	n/a		
Unanswered	6 (11%)	n/a		

Most participants expressed only mild to moderate concern regarding contracting influenza, which was paralleled by good self-reported health status and relatively low hospital admission rate (Table 2). In general, participants regarded the influenza vaccine favorably; 78% felt it was useful, and 82% deemed it to be safe (Table 2). Patients understood that influenza was not rare (84%), could be serious (80%), and was vaccine-preventable (69%; Table 3). Patients also correctly identified other vaccine-preventable diseases (Table 3). Furthermore, most participants (93%) recognized that persons over 65 years of age and younger adults with chronic medical conditions were at risk of more severe illnesses from influenza and correctly recognized that healthy children (45; 82%), and young adults without chronic conditions (50; 91%) were not in the same risk category. Pregnant women are deemed an at-risk group; only 34% of our population identified this correctly [25].

Six (11%) respondents live alone, 33% have one co-habitant, 22% have 2, and 34% have 3 or more co-habitants (Table 4). Despite excellent personal vaccine compliance and apparent influenza awareness, just over half (57%) of those surveyed were aware that seasonal influenza vaccination is recommended for co-habitants. Most of this group (73%) stated specific advice came from a healthcare source (Table 4). There was a positive correlation between receiving advice regarding vaccination of co-habitants and uptake in at least one household member (chi-squared, *p* < 0.001). Unfortunately, overall household vaccination rates were suboptimal. Of the 49 participants (89%) that live with at least one other person, less than half (48%) reported vaccination of one or more household members. Twelve (24%) had vaccine coverage of all household members (Table 4).

**Table 4:** Vaccination of co-habitants. Most participants had at least one household member. Over half were aware that vaccination is indicated for co-habitants, but less than one quarter had full vaccine cover at home. Awareness about vaccination for co-habitants was correlated with increased uptake. Chi-squared results are represented by *p* values, *p < 0.05

**Household contacts**					
Live Alone	6 (11%)				
1	18 (33%)				
2	12 (22%)				
≥ 3	19 (34%)				
**Vaccination of co-habitants**					
	***N*** **(%)**	**All co-habitants**	**Some co-habitants**	**None**	
Have co-habitants	49 (89%)	12 (24%)	n/a	25 (51%)	
Lives with 1	18 (37%)	8 (44%)	n/a	10 (59%)	
Lives with ≥ 2	31 (63%)	4 (13%)	12 (39%)	15 (48%)	
**Aware that cohabitants should be vaccinated**					
			**≥ 1 Co-habitant vaccinated**		
		***N*** **(%)**	**Yes (%)**	**No (%)**	**Chi-squared**
Aware of vaccination		28 (57%)	22	6	< 0.001*
Not aware		21 (43%)	1	20	
**Information source**					
Informed by healthcare worker		19 (68%)			
Informed by non-healthcare worker		7 (25%)			
Unanswered		2 (7%)			

## Discussion

The seasonal influenza vaccine is the most effective way of preventing influenza disease, and annual vaccination is recommended to close contacts of high-risk populations to limit transmission [3]. Despite potential disease severity, availability of an effective vaccine, and public campaigns to encourage uptake, compliance remains low in many susceptible groups [21, 22].

Patients with hypogammaglobulinemia are deemed at high risk of infective complication, in spite of adequate immunoglobulin replacement therapy [17, 27]. Those with antibody deficiency might benefit modestly from vaccination and may rely greatly on herd immunity for optimal health outcomes [28]. The focus of this study was to assess seasonal influenza vaccine in patients and co-habitants of those receiving IgRT. We report a reassuring 76% uptake of the influenza vaccine during the 2019/20 season, which meets the WHO target coverage rate for at-risk groups [4] (Table 2). Compliance was 73% in those < 65 years, exceeding uptake rates of aged-matched patients with other chronic diseases in numerous developed nations [21–24]. This was a single-center study with a small sample size; however, our results are in keeping with two North American surveys for the 2016/17 season. One reported 75% uptake among one thousand adults with a primary antibody deficiency; the other reported 76% in over 600 patients with CVID [25, 29]. Neither study investigated patient insight or vaccination of co-habitants.

In our study, vaccination rates were consistent between age groups, but there was a sex bias; 92% compliance among females, compared with 65% in males (chi-squared, *p* < 0.05; Table 1). The reason for this is unclear. A gender difference in vaccine uptake is not universally reported. International data is mixed; many report no difference, several studies find men are more likely to receive vaccination than women, and others report the reverse [30–32].

Previous studies have shown certain factors are associated with increased vaccine uptake, knowledge that influenza is common and can be serious, trust in the safety of vaccination, and physician recommendation [21, 23, 24]. Many of these factors were identified in our study, and it is widely acknowledged that recommendation by a healthcare worker is one of the strongest modifiable factors to encourage vaccine uptake [33, 34]. Our data support the important role of physicians in vaccine advocacy, particularly concerning advice for vaccination of co-habitants (Table 4). Various studies report the misconception that the influenza vaccine can cause an acute influenza infection is a common barrier to vaccination [23]. This was not prevalent in our group.

Self-identification, awareness, and knowledge among patients receiving IgRT highlight an understanding that is often absent in other at-risk cohorts. Many perceived barriers to vaccination were not present, for example, mistrust or poor understanding of its role in preventing disease [22, 23] (Table 3). Interestingly, population-based research indicates that the inability to self-identify as “at-risk” is a major barrier to vaccine uptake, particularly in those < 65 years of age [21, 22, 35]. IgRT is a relatively intrusive therapy, and historically, patients have a high rate of healthcare attendances, thus allowing abundant opportunity for patient education. Furthermore, frequent interactions could foster self-identification as being susceptible to infection, making patients more cautious in seeking vaccination [36]. We did not record the duration of IgRT for each participant, so we are unable to specifically comment on frequency of healthcare interactions. Over two-thirds of our participants have access to free public healthcare through a medical/GP card. Correlation between medical/GP card ownership and vaccine uptake was significant (chi-squared < 0.05; Table 2). Similarly, previous Irish data found that having free healthcare was a positive predictor for vaccination [21]. However, when our population was asked specifically about cost incurred by vaccination, there was no significant difference in vaccine uptake between those who paid compared with those who were vaccinated for free (chi-squared, *p* = 0.22; Table 1). Medical card ownership and increased vaccine uptake are likely multifactorial and not solely based on availability of free vaccination. Cost as a barrier to vaccination is deemed by many physicians to be a foregone conclusion; however, it is an area that merits further scrutiny. Interestingly, a population-based survey in North America demonstrated a divergence in opinion between patients and healthcare providers in this regard. In a setting where access to publicly funded healthcare is limited, a minority of patients viewed cost as a barrier to vaccination, whereas up to two-thirds of physicians believed that monetary concerns were a primary deterrent [35, 37]. As countries strive toward optimal vaccine coverage, efforts are continually being made to identify barriers for engagement. These results emphasize that education and awareness of the influenza vaccine may surpass access to free vaccination; as one is less likely to seek immunization, if unaware of its benefit, regardless of cost.

There was a low rate of vaccine uptake in co-habitants; only 12 participants (24%) had full vaccine cover at home. This study shows a correlation between advice for vaccination of co-habitants and rate of coverage in household members (chi-squared, *p* < 0.001; Table 4). Similar rates of vaccination coverage have been documented by others that assessed household members of those with chronic disease [38]. The age, risk profile, and health of co-habitants were not recorded in our data; thus, we are unable to discriminate between household members that were vaccinated to protect our patient, or for another reason. Nonetheless, these findings do highlight a significant gap in vaccine coverage that exposes our cohort to increased transmission risk. This could be modifiable with focused education regarding the importance of vaccination in all close contacts.

Vaccination of healthy household members may be perceived as challenging. However, data show that decreasing transmission to vulnerable individuals is a strong motivation for healthy adults [37]. In a university-based survey, 71% of unvaccinated healthy students indicated willingness to be vaccinated in light of helping others [37]. These findings emphasize that individuals can be incentivized to engage in vaccination uptake for altruistic means.

### Study Strengths and Limitations

This is a small, single-institution study; thus, some findings may not be generalizable to other populations. A response rate of 57% exceeds many population-based questionnaires [24, 29], and 76% influenza vaccine uptake parallels findings from larger international surveys in a similar population [29, 34, 35]. High educational attainment was noted in participants, 62% holding a tertiary level qualification, which in theory could self-bias toward active engagement with healthcare information. Contrary to this, others have demonstrated that educational level is not a self-determining predictor of vaccine uptake [39, 40].

The questionnaire was extensive and previously validated. Self-selection of those in favor of vaccination could predispose to engagement in the questionnaire. There is no national registry for influenza vaccination to allow us to compare our vaccination rate with an objective measure. However, many studies on self-reported influenza vaccine status have established robust validity in a questionnaire-based approach [25, 40]. Moreover, data collection took place immediately after the vaccination season, thus limiting recall bias.

## Conclusion

This study shows satisfactory influenza vaccine uptake among patients receiving IgRT in a large referral hospital. Our population meets WHO target vaccine coverage, parallels international findings, and greatly exceeds influenza immunization in other high-risk groups [21, 25, 29]. The majority of patients had previously received advice on seasonal vaccination, felt well-informed, and stated a physician as their primary information source.

Vaccine uptake among household members was low, thus potentially increasing influenza transmission risk. To our knowledge, this is the first time that this has been reported in an immunodeficiency cohort. This study demonstrates a need to specifically recommend immunization to close contacts and household members of these patients. Enhancing vaccination rates in co-habitants to those at-risk is likely achievable. In light of the current study, we recommend healthcare workers emphasize the importance of an annual influenza vaccine to patients on IgRT and educate them that vaccination of close contacts offers additional protection. For optimal impact, this advice should be teamed with complementary written information. A follow-up study is being planned to assess the outcome of this intervention and further delineate vaccination uptake rate of co-habitants.
